# Elevated expression of FREM1 in breast cancer indicates favorable prognosis and high‐level immune infiltration status

**DOI:** 10.1002/cam4.3543

**Published:** 2020-10-14

**Authors:** Han‐ning Li, Xing‐rui Li, Zheng‐tao Lv, Miao‐miao Cai, Ge Wang, Zhi‐fang Yang

**Affiliations:** ^1^ Department of Thyroid and Breast Surgery Tongji Hospital Tongji Medical College Huazhong University of Science and Technology Wuhan Hubei China; ^2^ Department of Orthopedics Tongji Hospital Tongji Medical College Huazhong University of Science and Technology Wuhan Hubei China; ^3^ College of Life Sciences and Health Wuhan University of Science and Technology Wuhan Hubei China

**Keywords:** biomarker, breast cancer, FRAS1‐Related Extracellular Matrix 1 (FREM1), immune infiltration, prognosis

## Abstract

Breast cancer (BC) poses one of the major threats to female's health worldwide. Immune infiltration in BC is a key representative of the tumor microenvironment and has been proven highly relevant for prognosis. The role of the FREM1 (FRAS1‐Related Extracellular Matrix 1) gene in carcinoma has not studied, moreover, the underlying mechanism remains largely unknown. This study aims to investigate the expression profile and potential action of FREM1 on BC progression. We applied series of bioinformatic methods as well as immunohistochemistry (IHC) and immunofluorescence (IF) to analyze FREM1 expression profile, its relationship with clinicopathological characteristics, impact on clinical outcomes, relevant functions, correlation with immune infiltration in BC. The results demonstrated that FREM1 had a dramatically reduced expression in BC tissues, possessed an inverse correlation with stage, age, and metastasis, and exhibited a higher level in invasive lobular breast carcinoma than in ductal one. Furthermore, decreased FREM1 expression was often associated with estrogen receptor (ER)/progesterone receptor (PR) negative and triple negative breast carcinoma (TNBC) status while human epidermal growth factor 2 (Her‐2) positive status, and considerably correlated with a worse overall survival (OS) and recurrence‐free survival (RFS). Meanwhile, the univariate/multivariate Cox model revealed that low‐FREM1 expression can be an independent prognostic factor for BC. Additionally, FREM1 was mainly involved in the cell metabolism and immune cells infiltration. Moreover, IHC and IF demonstrated a positive correlation of its expression with the immune infiltrating levels of CD4^+^, CD8^+^ T cells, and CD86^+^ M1 macrophages while a negative correlation with CD68^+^ pan‐macrophages and CD163^+^ M2 macrophages. These findings suggest that FREM1 can be a potential biomarker for evaluating the immune infiltrating status, and the BC prognosis.

## Graphical abstract

This study aims to investigate the expression profile and potential action of FREM1 on BC tumorigenesis. We applied series of bioinformatic and statistical methods to analyze FREM1 expression profile, its diagnostic efficiency, relationship with clinicopathological characteristics, impact on clinical outcomes, relevant functions, correlation with immune infiltration in BC. We provide the first evidence that increased FREM1 expression correlates with favorable clinical outcomes and mediates the regulation of the function and recruitment of immune cells.

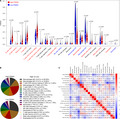

## 1 INTRODUCTION

Breast cancer (BC), the malignant condition with the highest incidence worldwide, is one of the leading causes of cancer‐related death in women worldwide. It occupies ~24% of all new cancer cases in women, with the occurrence of 2.08 million patients and ~627,000 mortalities annually.^1^ The BC incidence in China ranks first among female malignancies, accounting for 7% to 10% of systemic malignancies, and displays an upward and younger trend year by year.^2^ The occurrence, development and prognosis of BC are complex processes, involving numerous genes and proteins.^3^ Although a few advances have been achieved in targeted therapies, such as Trastuzumab, a drug targeting the Human Epidermal Growth Factor Receptor‐2 (Her‐2), some patients would still acquire drug resistance and develop tumor progression due to the widespread molecular heterogeneity of BC.^4^ Therefore, a novel target for the treatment and the identification of prognostic markers are of great significance for reducing BC patient's mortality.

FREM1 (FRAS1‐Related Extracellular Matrix 1), together with FRAS1 and FREM2, belonging to the FRAS/FREM extracellular matrix protein system, are series of extracellular matrix (ECM) proteins. They play a vital role in mediating the adhesion process between the epidermal basement membrane and the subdermal layer during embryonic development.^5^, ^6^ Notably, recessive mutations in the coding region of FREM1 can cause a number of human abnormal conditions, including Manitoba oculotrichoanal syndrome (MOTA), Fraser syndrome, and bifid nose/anorectal and renal anomalies syndrome (BNAR).^6^, ^7^, ^8^, ^9^ Under physiological circumstances, FREM1 is widely expressed in epithelial‐mesenchymal interaction and epidermal remodeling areas, for example, hair follicles, sensory vibrissae, teeth, footpads, and breast tissue.^10^ Growing studies have shown that ECM‐related proteins may modulate the migration and invasion of cancer cells through related signaling pathways, such as β1‐integrin‐Src‐EGFR,^11^ MAPK‐YAP,^12^ and PI3K‐AKT signaling.^13^ More importantly, the deposition, reconstruction, and cross‐linking of ECM can reprogram the local microenvironment and regulate the pro‐ and antitumor immune responses upon the stimulation of different ECM‐related proteins.^14^ Indeed, a previous study has reported that knockdown of FRAS1 (one of the numbers of the FRAS/FREM family) inhibited cell migration and invasion through regulating FAK signaling in lung cancer cell line A549.^15^ In spite of these, it remains unknown whether FREM1 is involved in the progression of cancers and whether its expression in BC is related to clinical outcomes and immune infiltration.

Here, we performed an integrated bioinformatic analysis to assess the expression pattern, prognostic value, possible molecular mechanism of FREM1 in immune cell infiltration of the BC progression. Besides, we also validated its expression and the relationship of it with infiltrating immune cells by IHC and IF on BC specimens. Our findings reveal the vital role of FREM1 in BC, provide an underlying association of FREM1 with tumor–immune interactions, as well as illustrate a potential mechanism for it.

## 2 MATERIALS AND METHODS

### 2.1 Data acquisition and patient characteristics

The transcriptomic data from RNA‐seq and paired clinical information (such as gender, age at initial diagnosis, histological types, clinical stage, tumor‐node‐metastasis (TNM) stage, classical breast cancer molecular markers (estrogen receptor [ER], progesterone receptor (PR), and Her‐2) with breast cancer were retrieved from The Cancer Genome Atlas (TCGA) database (https://tcga‐data.nci.nih.gov). Demographic and clinical characteristics of patients are shown in Table S1. FREM1 mRNA expression values (Reads Per Kilobase Million, FPKM) were extracted from the raw data, and the log2 transformed FPKM values (log2 [FPKM + 1] were calculated.

In addition, we downloaded microarray series of GSE71053,^16^ GSE120129,^17^ GSE42568,^18^ GSE29431, and GSE50567 ^19^ from the Gene Expression Omnibus (GEO) database at NCBI (https://www.ncbi.nlm.nih.gov/geo/). The information of these series is detailed in Table S2.

Moreover, the average methylation level on the FREM1 promoter region based on TCGA breast cancer data set was investigated by querying the UALCAN portal (http://ualcan.path.uab.edu).^20^ Methylation data of BC tissues and adjacent non‐tumoral tissues were calculated in units of beta values in accordance with the level of methylation ranging from 0 (unmethylated) to 1 (fully methylated).

### 2.2 Survival analysis of FREM1

The web tool Kaplan–Meier plotter (KM‐plotter; http://kmplot.com/analysis/) is a comprehensive database for evaluating the effect of specified genes on the survival of a lot of cancers including breast cancer. This system includes RNA‐seq and gene chip data sources from GEO, TCGA, and EGA databases.^21^ In the present study, KM‐plotter was utilized to evaluate the prognostic value of FREM1 expression in patients with breast cancers based on Affymetrix gene chip. In order to analyze the overall survival (OS), recurrence‐free survival (RFS), and distant metastasis‐free survival (DMFS), these individuals were first divided into two groups in line with the median level of FREM1 expression. The results were presented as Kaplan–Meier plots with hazard ratios (HR) and *p*‐values from a log‐rank test.

In addition, Kaplan–Meier survival curves were constructed using TCGA breast cancer cohort to evaluate the impact of FREM1 on OS and RFS of patients with different clinical characteristics (e.g., age, clinical stage, and molecular subtype). A *p* < 0.05 was regarded as significant for the above analyses.

### 2.3 Gene set enrichment analysis (GSEA)

To further elucidate the function of FREM1 in the pathogenesis of BC, GSEA which is a computational pathway analysis tool for determining whether the behavior of a predefined gene set shows statistical significance and concordant differences between two biological phenotypes (http://www.broadinstitute.org/gsea/index.jsp)^22^ was performed using the above‐mentioned breast cancer data sets from TCGA. In the current study, BC samples were sorted into low to high levels according to their relative FREM1 expression values, with defining the first third as the low‐FREM1 expression group and the last third as the high‐FREM1 expression group. Next, GSEA was performed using GSEA v4.0.1. The following gene sets, including Hallmarks (h.all.v7.0.symbols.gmt) and Kyoto Encyclopedia of Genes and Genomes (KEGG) pathway (c2.cp.kegg.v7.0.symbols.gmt), were utilized to identify and elucidate specific and predefined biological processes or states. The number of permutations for each analysis was set to 1000 and the permutation type was set at “phenotype,” and other parameters were set to the default. To quantify significant enrichment, the statistical threshold was set to the nominal *p* < 0.01 combined with false discovery rate (FDR) < 0.05.

### 2.4 Assessment of tumor‐infiltrating immune cells

To evaluate the effect of FREM1 expression on the tumor immune microenvironment, the proportion of 22 types of immune cells infiltrated in BC was estimated by using Cell‐type Identification By Estimating Relative Subsets Of known RNA Transcripts (CIBERSORT; http://cibersort.stanford.edu/), a deconvolution algorithm web tool for calculating and gauging the composition of immune cells based on high‐throughput sequencing data.^23^ Here, we used the TCGA breast cancer data set and ranked the gene expression matrix of 1109 cancer samples according to the expression level of FREM1. Samples with the top third and the bottom third FREM1 expression were correspondingly categorized into high‐FREM1 and low‐FREM1 expression groups, respectively. Meanwhile, we also explore the effect of FREM1 on the distribution of immune cells in BC patients with different molecular subtypes (Luminal, Her‐2 overexpression and triple negative breast cancer [TNBC]). For subgroup analysis, the same grouping procedure was used as above. The sorted data set was then uploaded to the CIBERSORT portal, with choosing the algorithm running with immune cell subtypes (LM22) signature and 1000 permutations. *p*‐values for deconvolution were generated via Monte Carlo sampling, which allows CIBERSORT to test the null hypothesis that no immune cell subtypes are existed in a given gene expression profile and establishes a measurement of accuracy of the results. The simples with a CIBERSORT output of *p* > 0.05, representing a greater proportion of nonimmune cells within the tumorous tissues, were excluded from the cohort. A flow chart describing the principal steps of CIBERSORT is provided in Figure S1. Violin plots were applied to visualize the distribution of immune cells among groups in accordance with the CIBERSORT results, while the differences were examined by the Wilcoxon test. *p* < 0.05 was regarded as statistically significant. Furthermore, the correlation heatmap was drawn to display the correlations of 22 types of infiltrated immune cells in BC tissues. The Pearson correlation coefficient was utilized to compute the correlation among different cell types. Absolute values of rho(*r*) reached 0.3–0.5 were categorized as weak correlations, 0.5–0.8 were moderate correlations, and >0.8 were strong correlations.

### 2.5 Tissue samples, immunohistochemistry (IHC), and immunofluorescence (IF)

A retrospective simple collection from Tongji Hospital, Tongji Medical College of Huazhong University of Science and Technology (Wuhan, China) of 30 pathology‐confirmed BC paraffin‐embedded (FFPE) tissues was carried out. The patients included in the study were Chinese females, aged between 24 and 71 years (median age, 55 years) and had not received radiotherapy, chemotherapy, targeted therapy, or any other treatment before surgery. A signed informed consent was obtained from each participant and the experimental protocol was granted by the Ethics Committee of Tongji Hospital, Tongji Medical College of Huazhong University of Science and Technology.

For IHC, FFPE tissues were sliced into 4‐μm‐thick sections, dewaxed with gradient alcohol, followed by antigen retrieval using sodium citrate repair buffer (PH = 6.0). Sections were incubated with primary antibodies, including rabbit antihuman FREM1 (Proteintech, 13086‐1‐AP, dilution ratio = 1:50), rabbit antihuman CD4 (Abcam, ab133616, dilution ratio = 1:500), rabbit antihuman CD8 (Abcam, ab4055, dilution ratio = 1:200), and rabbit antihuman CD68 (Abcam, ab125212, dilution ratio = 1:100) overnight at 4°C, followed by color development with HRP/DAB IHC detection kit (Abcam, ab64261). For immunophenotyping assessment, CD4 and CD8 were utilized as markers of T lymphocyte subtypes, CD68 was considered as a pan‐macrophage marker.^24^ The sections were examined using a Nikon digital camera (DS‐U3; Nikon, Japan; magnification, ×100) mounted on a Nikon microscope (Eclipse C1) by two experienced pathologists. The evaluation of FREM1 expression were scored in accordance with dyeing intensity and positive cell area ratio on a 3‐ and 4‐point scale. Dyeing intensity was graded as follows: 0, none of the cells scored positively; 1, weak staining; 2, moderate staining; or 3, strong staining. The positive cell area ratio was scored as follows: 0, no staining; 1, ≤25% of cells were stained; 2, 26%–50% of cells were stained; 3, 51%–75% of cells were stained; and 4, >75% of cells were stained. The final staining index was calculated as the sum of the intensity and percentage scores. Additionally, high‐FREM1 expression was defined as IHC score ≥ 4, while low‐FREM1 expression was defined as IHC score < 4. The extent of infiltrating CD4^+^ cells, CD8^+^ cells, and CD68^+^ cells in BC tissues was evaluated by direct cell count using Image J software (Version 1.47) on five different fields (magnification, ×100).

Furthermore, we performed double‐stained IF to detect the M1‐ and M2‐polarized macrophages in BC tissues with different FREM1 expression level. In this work, CD86^+^ cells were considered as M1‐polarized macrophages, whereas CD163^+^ cells were considered M2‐polarized macrophages.^24^ The BC sections after deparaffinization and rehydration procedures were boiled in EDTA (ethylene diamine tetraacetic acid) buffer (pH = 8.0) for epitope retrieval, followed by permeabilization and blocking with 0.1% of Triton‐X100 and 5% of BSA (bovine serum albumin) in PBS (Phosphate buffer saline) buffer for 30 min. Slides were then incubated with mouse antihuman CD86 primary antibodies (Abcam, ab270719, 2 µg/ml) for 2 h at room temperature. After extensively washing with PBS, the slides were incubated with Alexa Fluor‐647 anti‐mouse IgG (Abcam, ab150119, dilution ratio = 1:200) for 30 min. Slides were washed again with PBS and subject to rabbit antihuman CD163 (Abcam, ab87099, 5 µg/ml) for an additional 2 h, followed by incubated with Alexa Fluor‐488 anti‐rabbit IgG (Abcam, ab150077, dilution ratio = 1:200) for 45 min at room temperature. The sections were subsequently counterstained with DAPI (1 μg/ml, ServiceBio) for 5 min. Finally, the sections were sealed with the anti‐fluorescence quenching mounting medium and imaged using a Nikon fluorescent camera (DS‐U3; magnification, ×400) mounted on a Nikon microscope (Eclipse C1). CD86^+^ and CD163^+^ cells were calculated by cell count using Image J on five different fields.

### 2.6 Statistical analysis

Statistical analysis was carried out with R software (R Core Team, Version 3.5.1) or GraphPad Prism 7. The global differences of the FREM1 expression between the 113 normal tissues and 1109 breast cancer tissues were determined by the Wilcoxon test, whereas the differential expression between 113 paired donor‐matched normal and cancer samples was examined by the paired two‐tailed *t*‐test. Box plots were generated using “beeswarm” and “ggplot2” R packages. Logistic regression was applied to determine the association of clinical variables with the FREM1 expression. The dependent variables (FREM1 expression values) were dichotomized according to the median expression levels and the odds ratios (OR) and 95% confidence intervals (CIs) were decided for each specific clinical factor. Univariate and multivariate Cox proportional hazards regression analyses were conducted to determine clinicopathological parameters for OS and the results were expressed as hazard ratios (HR) and 95% CIs. The results of IHC and IF were present as means ± standard deviation (SD) or as scatter plots visualizing individual data values and were analyzed utilizing unpaired *t* tests. A value of *p* < 0.05 was regarded as statistically significant for all above analyses.

## 3 RESULTS

### 3.1 Decreased expression of FREM1 in BC

**Figure 1:**
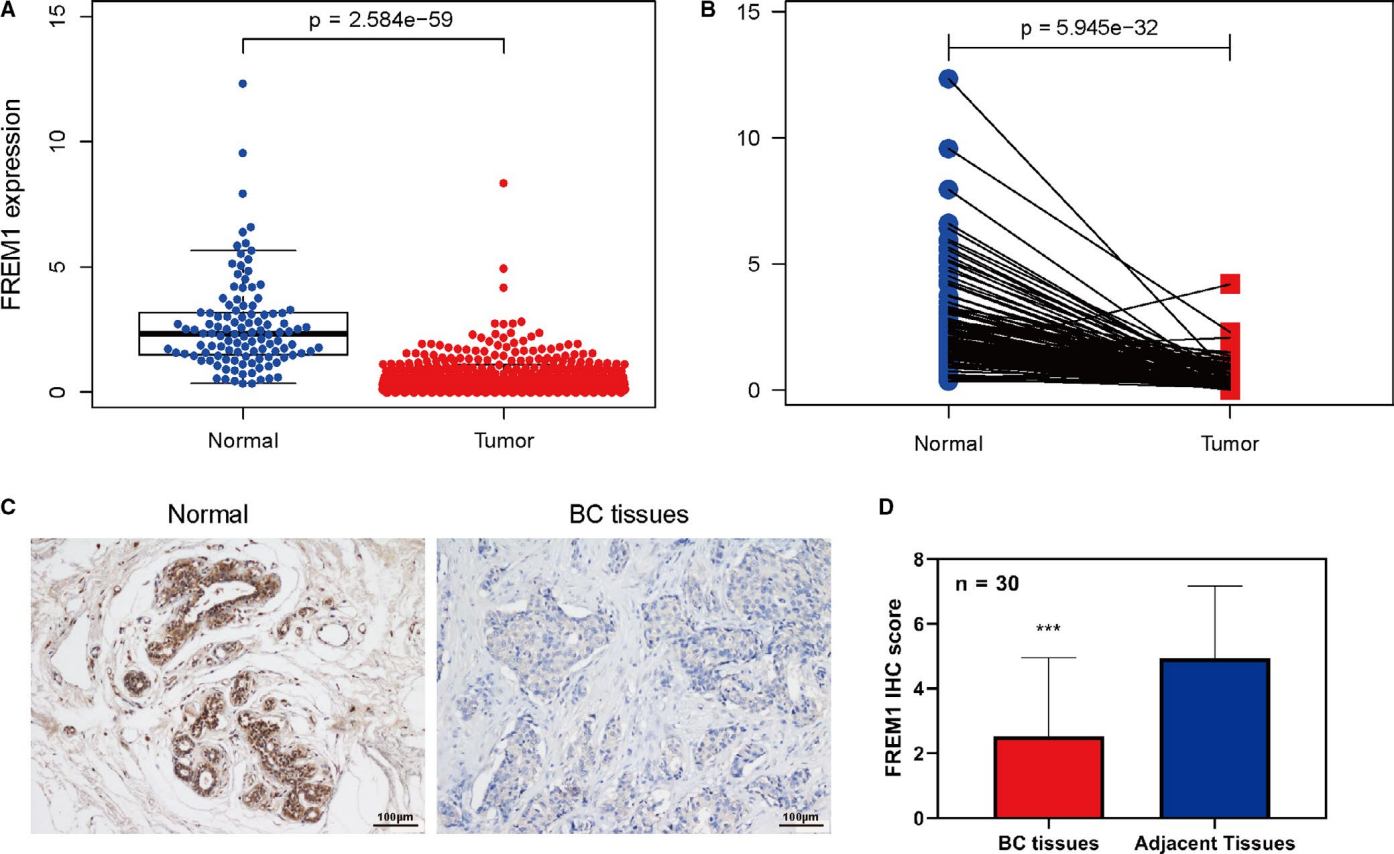
FREM1 expression level in BC. (A) The global differences of the FREM1 expression between the 113 normal tissues and 1109 breast cancer tissues in TCGA. (B) The differential FREM1 expression between 113 paired donor‐matched normal and cancer samples in TCGA. (C) IHC analysis of FREM1 protein in human BC and adjacent normal tissue specimens (*n* = 30). Representative images of FREM1 staining are shown. (D) FREM1 IHC scores are displayed in a histogram. ***p < 0.001. FREM1, FRAS1‐Related Extracellular Matrix 1; BC, breast cancer; TCGA, The Cancer Genome Atlas; IHC, immunohistochemistry

By analyzing TCGA data set, we found that FREM1 mRNA was significantly repressed in BC compared to that in normal tissue (*p* < 0.0001, Figure 1A). Moreover, the results from 113 paired cancer samples and adjacent tissues demonstrated that FREM1 mRNA level was dramatically downregulated in tumor tissues than in para‐cancerous tissues (*p* < 0.0001, Figure 1B). Consistent with these, the expression level of FREM1 was considerably reduced in BC tissues when compared to normal tissues in five GEO series (GSE71053, GSE120129, GSE42568, GSE29431, and GSE50567, all *p* < 0.01, Figure S2A–E). In addition, levels of FREM1 promoter methylation were remarkedly higher in BC than in normal tissue (*p* < 0.0001, Figure S2F). The lower expression of FREM1 was further confirmed by IHC in our cohort of BC patients (Figure 1C,D, *n* = 30). Collectively, these suggest that the transcription of FREM1 is decreased under the clinical status of BC.

### 3.2 Association between the FREM1 expression and the clinicopathological characteristics of BC

The logistic regression results summarized in Table 1 showed that the patients with elderly age (*p* = 0.001), advanced clinical stage (*p* = 0.047) and distant metastases (*p* = 0.038) had pronouncedly lower expression of FREM1. As for histological types, the FREM1 expression was dramatically higher in infiltrating lobular carcinoma (ILC) than that in infiltrating ductal carcinoma (IDC; *p* = 0.002). Furthermore, the patients having reduced FREM1 expression were much more frequently present as ER negative (*p* = 0.009), PR negative (*p* = 0.001), Her‐2 positive (*p* = 0.035), and TNBC status (*p* = 0.011). Taken together, these results demonstrate that patients with a lower FREM1 expression are more prone to develop to a more advanced stage than those with a higher FREM1 expression, suggesting a tight relationship of it with the clinicopathological characteristics of BC.

**Table 1:** Correlation of the FREM1 expression^a^ with the clinicopathological characteristics of breast cancer in accordance with the logistic regression analysis

Clinical characteristics	Total (*N*)	Odds ratio in FREM1 expression	*p*‐value
Age (≤50 vs. >50)	1097	1.56 (1.20–2.04)	0.001
Stage (I + II vs. III+IV)	1073	1.32 (1.00–1.75)	0.047
T (T1 + 2 vs. T3 + 4)	1094	1.37 (0.99–1.90)	0.057
N (N0 vs. N1 + 2 + 3)	1077	0.97 (0.76–1.23)	0.807
M (M0 vs. M1)	934	2.72 (1.11–7.64)	0.038
Histological type (IDC vs. ILC)	987	0.69 (0.53–0.97)	0.002
ER status (negative vs. positive)	984	0.68 (0.51–0.92)	0.009
PR status (negative vs. positive)	981	0.58 (0.44–0.75)	0.001
Her−2 status (negative vs. positive)	687	1.41 (1.03–1.96)	0.035
TNBC (non‐TNBC vs. TNBC)	719	1.56 (1.11–3.44)	0.011

Abbreviations: ER, estrogen receptor; Her‐2, human epidermal growth factor receptor‐2; IDC, infiltrating ductal carcinoma; ILC, infiltrating lobular carcinoma; PR, progesterone receptor; TNBC, triple negative breast cancer.

^a^ Categorical‐dependent variable, greater or less than the median FREM1 expression level.

### 3.3 FREM1 is an independent prognosis of superior survival in patients with BC

**Figure 2:**
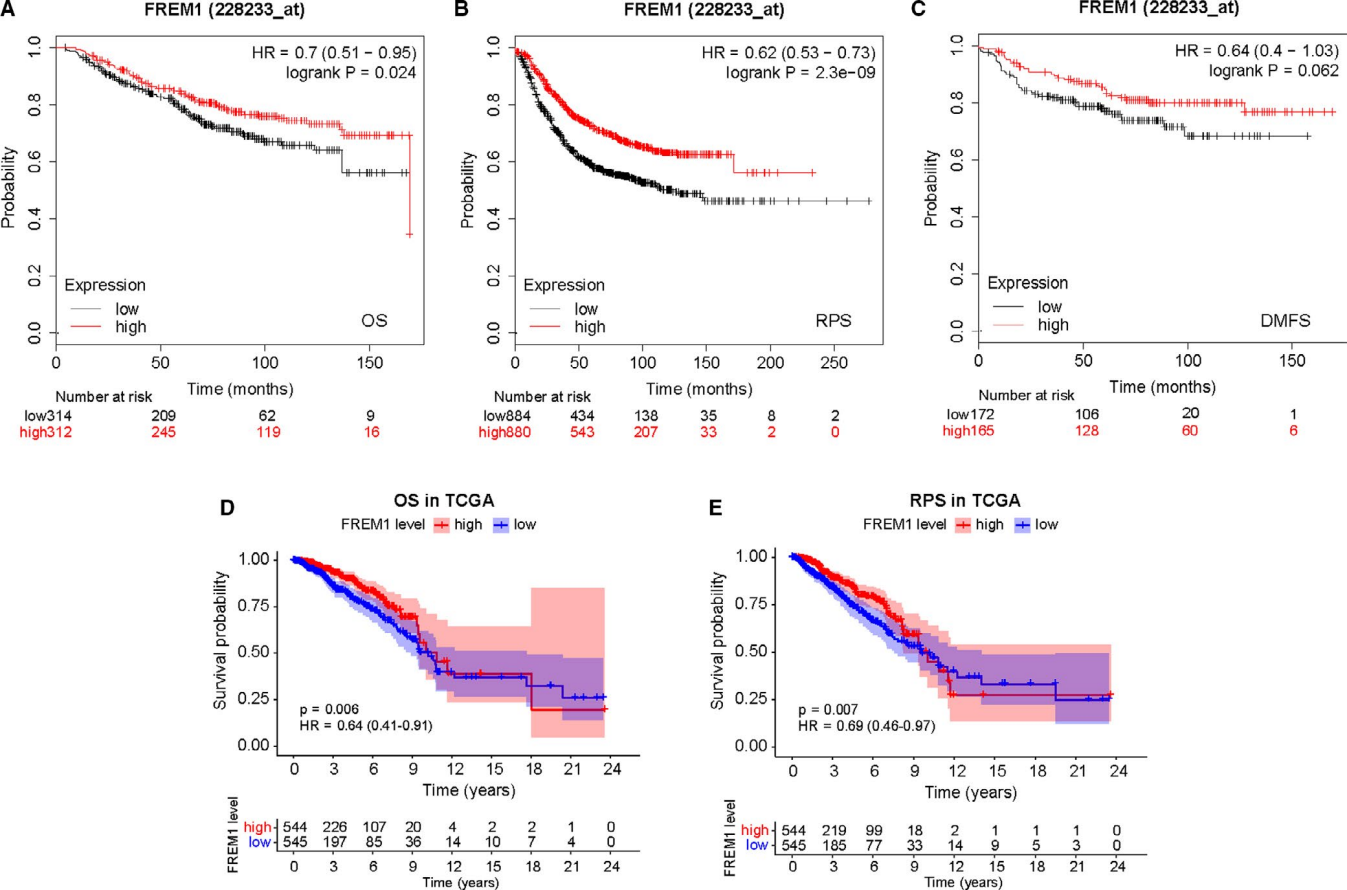
Associations of FREM1 mRNA expression and the prognosis of patients with BC. Kaplan–Meier curves based on the Kaplan–Meier plotter database for OS (A), RFS (B), and DMFS (C) of BC patients comparing the high‐ and low‐FREM1 expression levels. The impact of FREM1 mRNA expression on OS (D) and RFS (E) of BC patients according to data from TCGA. OS, overall survival; RFS, recurrence‐free survival; DMFS, distant metastasis‐free survival

**Figure 3:**
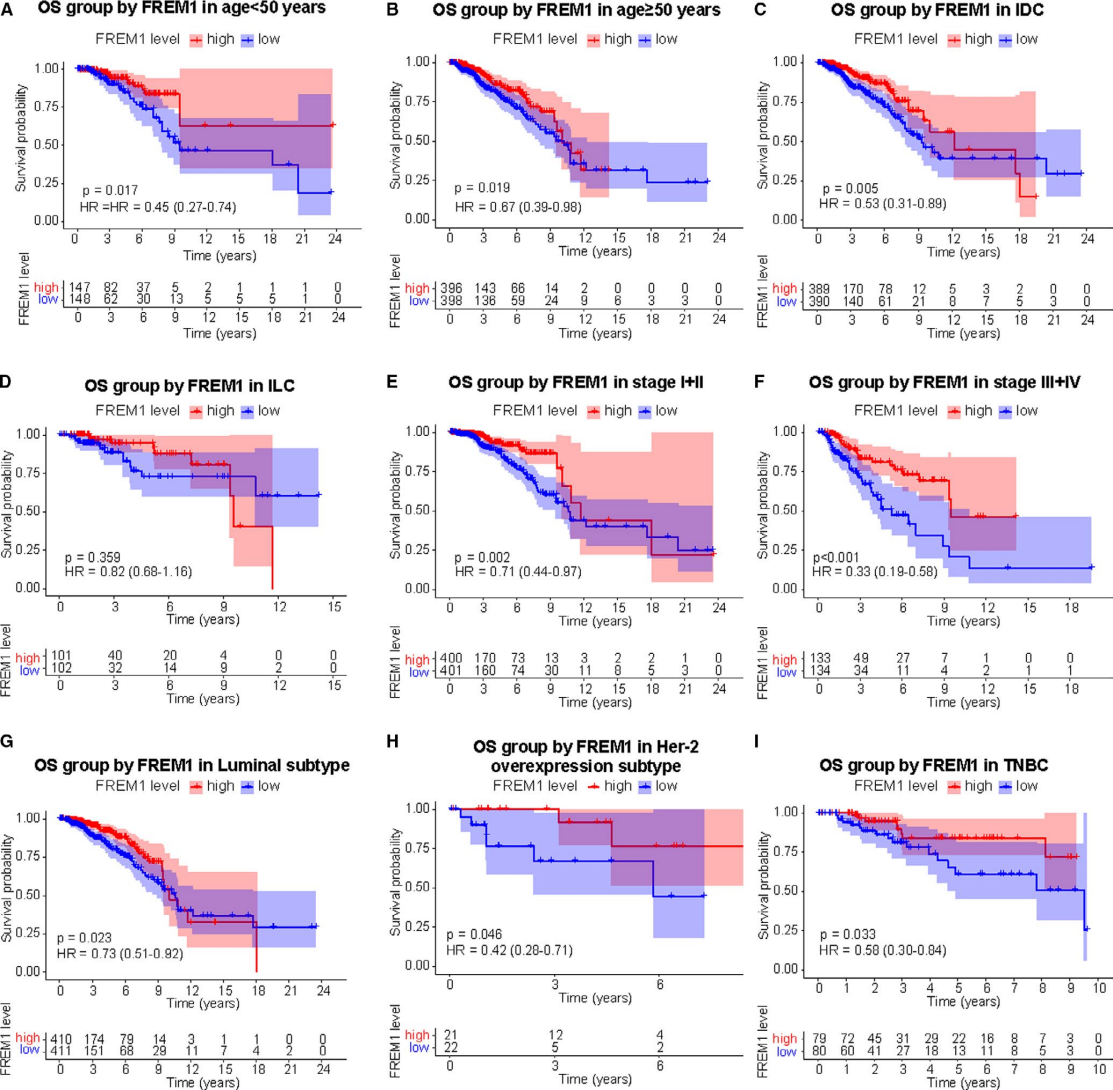
Effect of FREM1 mRNA expression on OS of BC patients stratified by different clinical features. Subgroup analysis of group age <50 years (A), ≥50 years (B), IDC (C), ILC (D), stage I/II (E), stage III/IV (F), Luminal (G), Her‐2 overexpression (H), and TNBC (I) subtypes. IDC, infiltrating ductal carcinoma; ILC, infiltrating lobular carcinoma; TNBC, triple negative breast cancer

**Figure 4:**
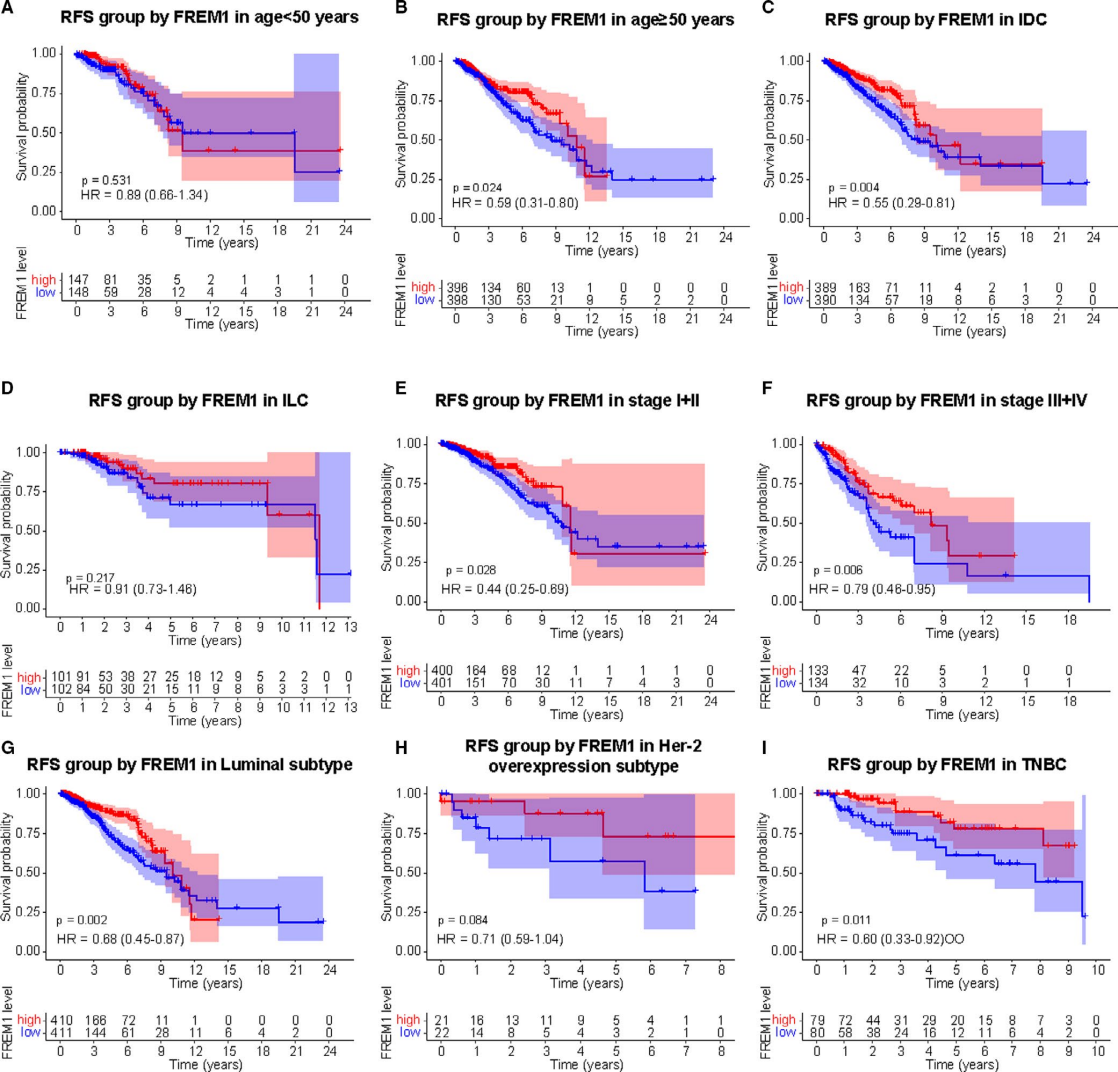
Effect of FREM1 mRNA expression on RFS of BC patients according to the different clinical characteristics. Subgroup analysis of group age <50 years (A), ≥50 years (B), IDC (C), ILC (D), stage I/II (E), stage III/IV (F), Luminal (G), Her‐2 overexpression (H), and TNBC (I) subtypes

As shown in Figure 2, Kaplan–Meier curves clearly indicated that reduced expression of FREM1 in patients was correlated with worse OS (HR = 0.7; 95% CI = (0.51–0.95); *p* = 0.024; Figure 2A) and RFS (HR = 0.62; 95% CI = (0.53–0.73); *p* = 2.3e‐09; Figure 2B). Moreover, lower FREM1 expression tended to be associated with shorter DMFS, with an obvious borderline significance (HR = 0.64; 95% CI = 0.4–1.03; *p* = 0.062; Figure 2C). Similar results were yielded with TCGA data, in where patients with low‐FREM1 expression exhibited shorter OS (HR = 0.64; 95% CI = 0.41–0.91; *p* = 0.006; Figure 2D) and RFS (HR = 0.69; 95%CI = 0.46–0.97; *p* = 0.007; Figure 2E) time than those with high‐FREM1 expression. Considering the heterogeneity of BC, we further performed OS and RFS analysis in subgroups of BC patients. The subgroup analysis demonstrated that patients with low‐FREM1 expression showed a worse outcome in group age <50 years or ≥50 years, IDC, stage I/II, stage III/IV, Luminal, Her‐2 overexpression, and TNBC subtypes, (Figure 3), and RFS was poor in the group with low‐FREM1 expression and an age ≥ 50 years, IDC, stage I/II, stage III/IV, Luminal, and TNBC subtypes (Figure 4). These results suggested that low expression of FREM1 can serve as a prognosticator of OS and RFS in subgroups of BC patients.

Univariate and multivariate Cox regression analyses were further performed on the TCGA breast cancer cohort to explore the factors influencing OS. Fantastically, the univariate Cox model revealed that age, clinical stage, T, N, and M classification, and FREM1 expression represented potential survival‐related factors (Table 2, left). Meanwhile, multivariate cox regression analysis of variable significance was conducted in the univariate assessment. Since T, N, and M classification show overlaps of information with clinical stage, we chose to include only the latter in the multivariate analysis. The results suggested that with the advancement of clinical stage and decreased FREM1 expression could also be independent risk factors for the OS of BC patients (Table 2, right).

**Table 2:** Univariate and multivariate Cox analyses of the associations between the prognostic signatures and the overall survival in breast cancer

Clinical variables	Univariate analysis			Multivariate analysis		
	HR	95% CI	*p*‐value	HR	95% CI	*p*‐value
Age (continuous)	1.57	1.06–2.31	0.024	1.47	0.99–2.11	0.064
Histological type (IDC vs. ILC)	1.03	0.82–1.98	0.712			
Stage (I + II vs. III + IV)	2.17	1.71–2.74	<0.001	1.98	1.09–2.87	0.015
T (T1 + 2 vs. T3 + 4)	1.54	1.25–1.92	<0.001			
N (N0 vs. N1 + 2 + 3)	1.70	1.41–2.05	<0.001			
M (M0 vs. M1)	4.28	2.60–8.45	<0.001			
ER status (negative vs. positive)	0.75	0.51–1.08	0.124			
PR status (negative vs. positive)	0.77	0.54–1.08	0.128			
Her‐2 status (negative vs. positive)	1.34	0.83–2.16	0.229			
TNBC (non‐TNBC vs. TNBC)	1.40	0.89–2.18	0.146			
FREM1 (low vs. high)	0.27	0.14–0.70	0.001	0.31	0.14–0.63	0.002

Abbreviations: ER, estrogen receptor; Her‐2, human epidermal growth factor receptor‐2; IDC, infiltrating ductal carcinoma; ILC, infiltrating lobular carcinoma; PR, progesterone receptor; TNBC, triple negative breast cancer.

### 3.4 Gene set enrichment analysis (GSEA)

**Figure 5:**
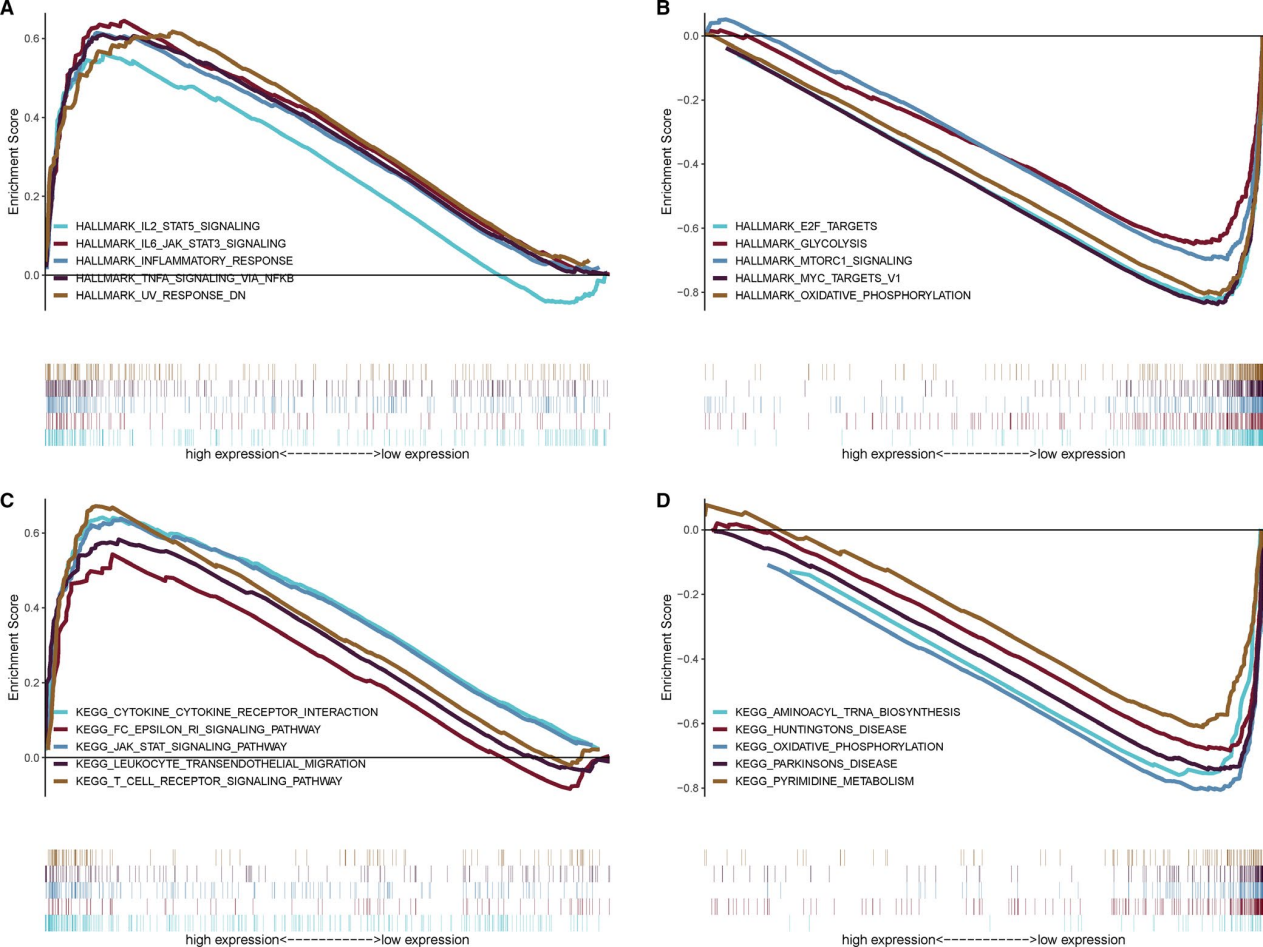
GSEA analysis for FREM1. GSEA using Hallmark and KEGG gene sets was performed to compare the high‐FREM1 and low‐FREM1 expression groups. The gene sets are sorted according to the absolute NES value, and the top five gene sets are displayed. The top 5 Hallmark gene sets enriched in the high‐FREM1 expression group (A) and the low‐FREM1 expression group (B). The top 5 KEGG gene sets enriched in the high‐FREM1 expression group (C) and the low‐FREM1 expression group (D). GSEA, gene set enrichment analysis; KEGG, Kyoto Encyclopedia of Genes and Genomes; NES, normalized enrichment score

To explore the potential signal pathways perturbed by FREM1 in BC, GSEA was applied to map into the Cancer Hallmarks and KEGG pathway databases. Seven statistically significant Hallmarks pathways, including UV‐response, IL2‐STAT5 signaling, inflammatory response, IL6‐STAT3 signaling, etc. enriched in high‐FREM1 expression group, were identified (Figure 5A; Table S3). It was worth mentioning that most enriched pathways were associated with the term immune system. Similarly, GSEA of low‐FREM1 expression group based on Hallmarks data set confirmed eight activated pathways involving cell metabolism (glycolysis, oxidative phosphorylation), cell‐cycle regulation (DNA repair, G2M checkpoint), and cancer‐related signaling (Myc‐targets, mTOR signaling, E2F targets; Figure 5B; Table S4).

Furthermore, GSEA on KEGG indicated that an abundance of pathways was enriched in the high‐FREM1 (23 pathways) and low‐FREM1 (26 pathways) expression groups, respectively (Tables S3 and S4). Similar to the results of Hallmarks, most important pathways enriched in the high‐FREM1 expression group pointed toward immune‐associated modulation, including JAK‐STAT signaling, cytokine‐cytokine receptor interaction, T cell receptor signaling, leukocyte transendothelial migration, etc. (Figure 5C; Table S3), while gene sets involved in cell metabolism (such as oxidative phosphorylation, pyrimidine metabolism, aminoacyl biosynthesis), genetic repair process (e.g., mismatch repair, base excision repair), and abnormal protein folding disorders (such as Huntington disease, Parkinson disease; Figure 5D; Table S4) were gathered around low‐FREM1 expression group. Therefore, these results strongly suggest that FREM1 participates in the regulation of multiple molecular signaling pathways in the progression of BC, especially in immune‐ and metabolism‐related pathways.

### 3.5 Association of FREM1 expression with tumor‐infiltrating immune cells in BC

**Figure 6:**
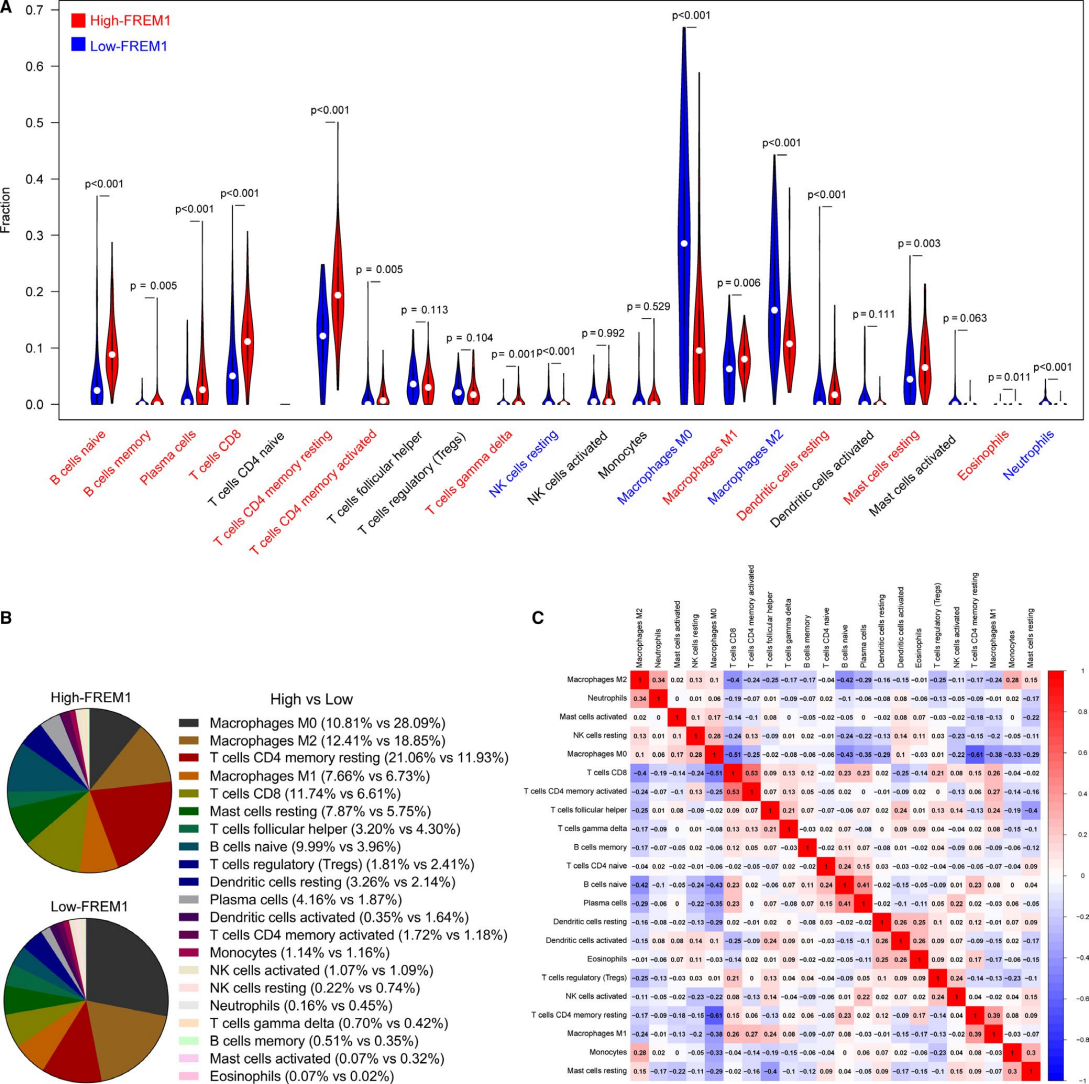
FREM1‐related immune cell infiltration alteration in whole cohort of BC. (A) Violin plots showing the distribution alteration of 22 types of immune cells between the high‐FREM1 and low‐FREM1 expression groups in whole cohort of BC. Compared to low‐FREM1 expression group, the deceased immune cell types are labeled as blue and the increased labeled as red (Wilcoxon test). (B) The relative proportions of 22 immune cell populations in high‐FREM1 and low‐FREM1 expression groups, respectively. (C) The correlation coefficient values between every two types of immune cells are represented in the form of heatmap in pairwise matrix (Pearson test)

Previous reports have indicated that the degree and proportion of immune cell infiltration have different impact on tumor prognosis.^25^, ^26^, ^27^ Based on the results of GSEA described above, we speculated a potential role of FREM1 in immune regulation. We therefore asked whether or to what extent FREM1 expression alters the distribution of immune cells within the local tumor microenvironment of BC. The proportion of 22 types of infiltrating immune cells was estimated by CIBERSORT. A total of 332 samples in low‐FREM1 expression group and 327 in high‐FREM1 expression group were eligible for evaluating immune infiltration according to Monte Carlo sampling (Figure S1). The results of CIBERSORT demonstrated that a considerable portion of the immune cells presents pronounced difference between the high‐FREM1 and low‐FREM1 expression groups, undoubtedly indicating a key regulator of FREM1 in tumor immune microenvironment. Specifically, subjects with a higher FREM1 expression tended to harbor higher proportion of B cells (naive and memory; *p* < 0.001 and *p* = 0.005, respectively), plasma cells (*p* < 0.001), CD8^+^ T cells (*p* < 0.001), CD4^+^ memory T cells (resting and activated; *p* < 0.001 and *p* = 0.005, respectively), gamma‐delta T cells (*p* = 0.001), M1 macrophages (*p* = 0.006), resting dendritic cells (*p* < 0.001), resting mast cells (*p* = 0.003), and eosinophils (*p* = 0.011; Figure 6A,B). Meanwhile, individuals with a lower expression of FREM1 were prone to proportionally have more abundance of M0 macrophages (*p* < 0.001), M2 macrophages (*p* < 0.001), resting natural killer cells (*p* < 0.001), and neutrophils (*p* < 0.001; Figure 6A,B). Of these, compared to low‐FREM1 expression group, the most markedly decreased cell type is M0 macrophages (28.09% vs. 10.81%, low vs. high, the same below) and the most obviously increased is resting CD4^+^ memory T cells (11.93% vs. 21.07%) in high‐FREM1 expression group. Additionally, a heatmap depicting the correlation coefficient between every two types of immune cells demonstrated some of the immune cell subpopulations exhibited a weak to medium correlation in BC tissues (Figure 6C). CD8^+^ T cells and activated CD4^+^ memory T cells present the strongest positive correlation (Pearson's *r* = 0.53), while M0 macrophages and resting CD4^+^ memory T cells exhibited the strongest negative correlation (Pearson's *r* = −0.61). Furthermore, M0 macrophages might act like a hub cell type, since the proportion of them negatively correlated with naive B cells, resting CD4^+^ memory T cells, CD8^+^ T cells, plasma cells, monocytes, and M1 macrophages (Figure 6C). These results collectively inferred that FREM1 may play a vital role in regulating the abundance of CD8^+^, CD4^+^ T cells, B cells, macrophages, mast cells, and dendritic cells.

### 3.6 Relationship between FREM1 expression and leukocyte representation in subtypes of BC

Owing to the high degree of heterogeneity at the molecular level, the three distinct subtypes of BC, namely Luminal (positive for ER and/or PR), Her‐2 overexpression (negative for ER and PR, positive for Her‐2), and TNBC (lack of ER, PR, and Her‐2 expression), have different biological, molecular, and clinical characteristics.^28^, ^29^ Thus, we further explored the effect of FREM1 on the leukocyte representation in these subtypes.

**Figure 7:**
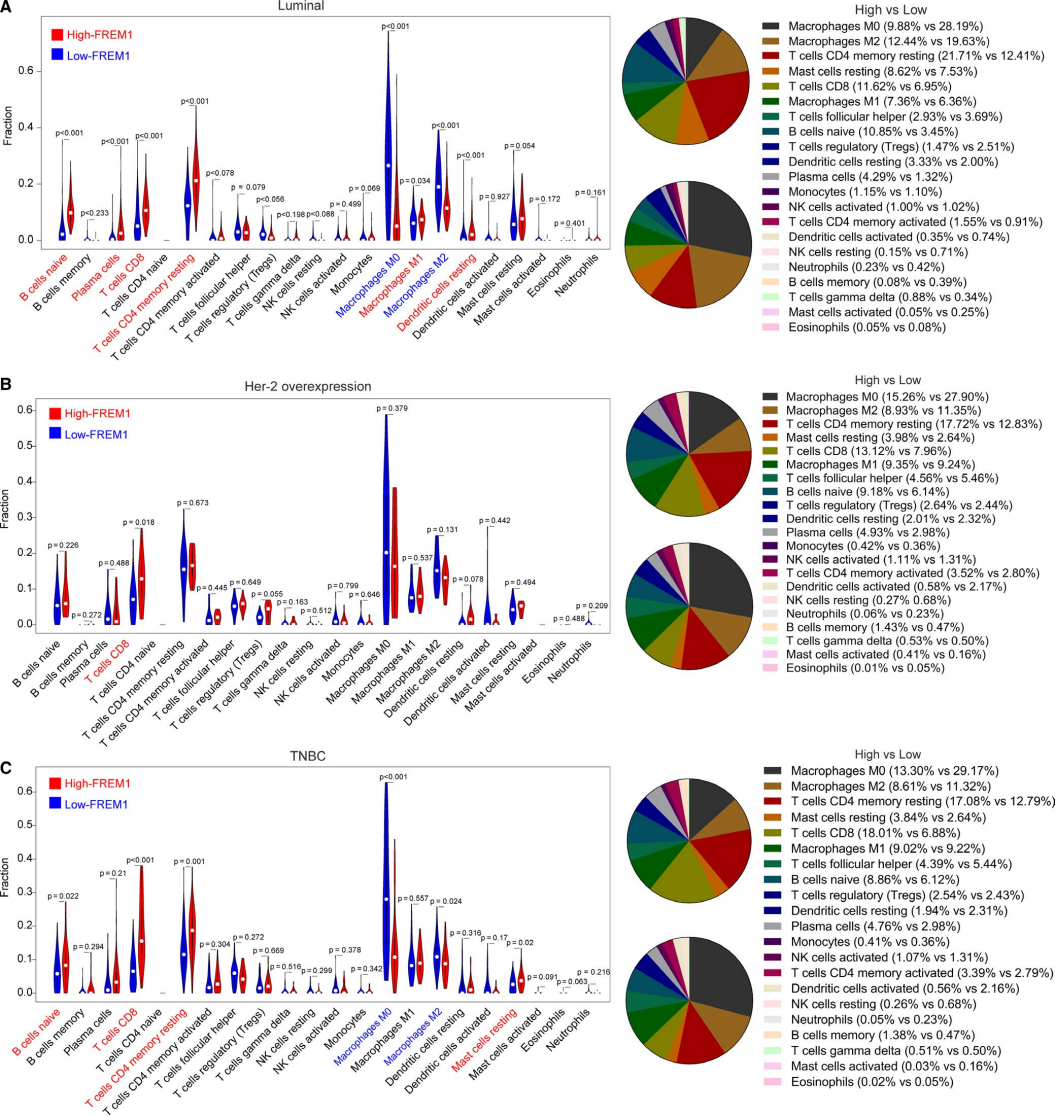
FREM1 expression and leukocyte representation in subtypes of BC. The distribution alteration of immune cells between high‐ and low‐FREM1 expression groups in Luminal (A), Her‐2 overexpression (B) and TNBC (C) subtypes

In Luminal subtype of BC, Figure S1 summarized the results obtained from 170 samples of low‐FREM1 expression group and 172 of high‐FREM1 expression group with a CIBERSORT *p* < 0.05. The differences in proportions of naive B cells, plasma cells, CD8^+^ T cells, resting CD4^+^ memory T cells, resting dendritic cells, and macrophages (M0/M1/M2) between low‐ and high‐FREM1 expression groups were statistically significant. Higher proportion for above significantly changed cell types existed in high‐expression group, compared with low‐expression group except for M0 and M2 macrophages (Figure 7A). This result is in agreement with that of Figure 6A,B, possibly due to the vast majority of BC cases belonging to this subtype.

As for Her‐2 overexpression subtype, 25 samples were filtered out with a CIBERSORT *p *< 0.05 (Figure S1). Of these, 12 samples were from low‐expression group and 13 samples were from high‐expression group. Surprisingly, only CD8^+^ T cells of 22 subpopulations of infiltrating immune cells was found to have statistically significant differences between high and low‐expression group (13.12% vs. 7.96%, high vs. low, *p* = 0.018; Figure 7B).

In TNBC subtype, 36 samples of low‐expression group and 34 samples of high‐expression group met screening criterion (Figure S1). Naive B cells, CD8^+^ T cells, resting CD4^+^ T memory cells, M0 macrophages, M2 macrophages, resting mast cells were affected by FREM1 expression. Among them, naive B cells (*p* = 0.022), CD8^+^ T cells (*p* = 0.004), resting CD4^+^ memory T cells (*p* = 0.001), and resting mast cells (*p* = 0.02) show a higher proportion in high‐FREM1 expression group compared with low‐FREM1 one. On the contrary, the proportion of M0 macrophages (*p* = 0.001), M2 macrophages (*p* = 0.024) are obviously lower (Figure 7C). Notably, the ratio changes of CD8^+^ T cells (18.01% vs. 6.88%, high vs. low) between low‐ and high‐expression group in TNBC are more significant than that of the other two types, which is worthy of further investigation.

### 3.7 Validation of correlation between FREM1 and immune cell composition from clinical specimens

**Figure 8:**
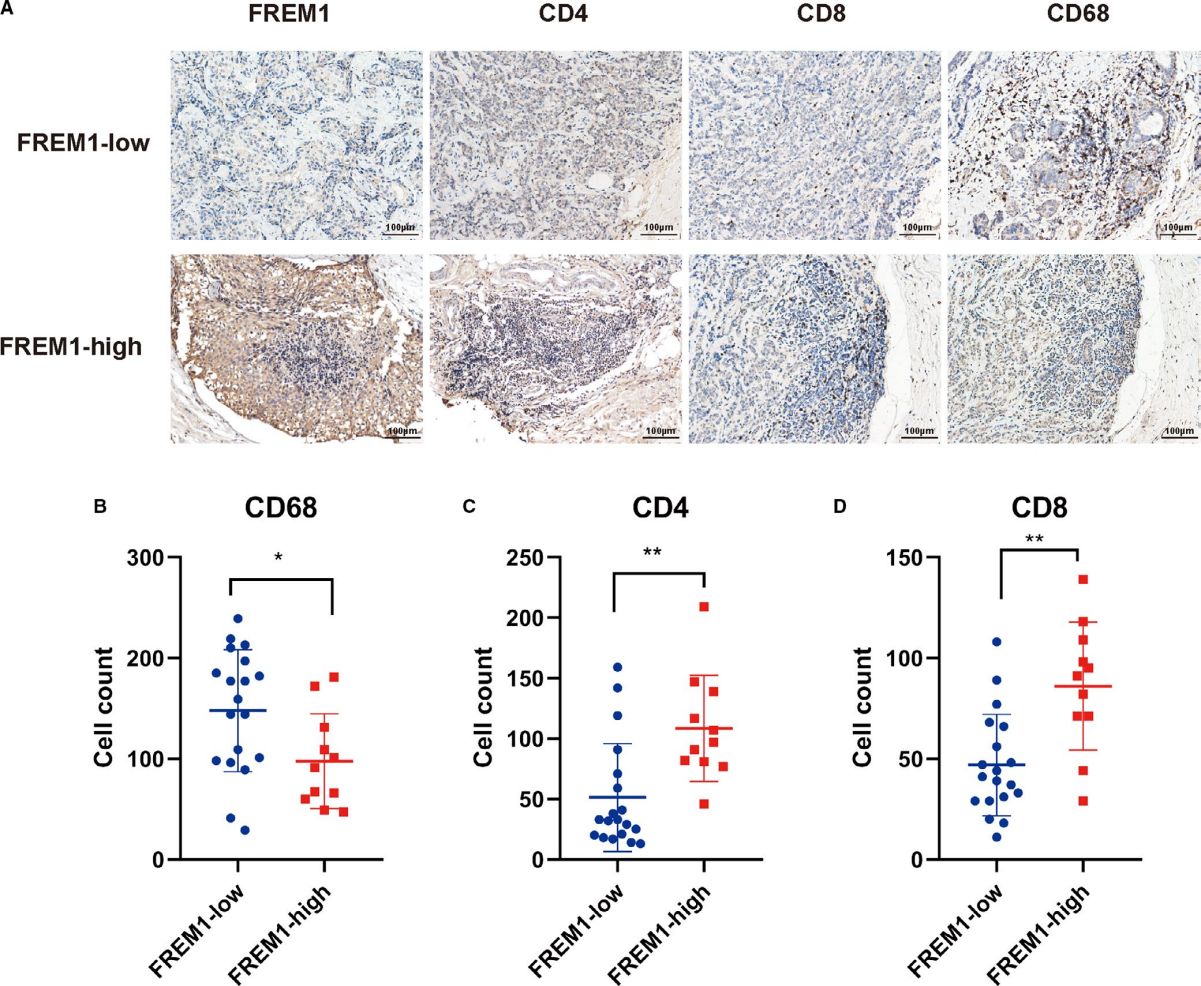
Immunohistochemical staining for CD4, CD8, and CD68 to detect T cells and macrophages infiltration in BC tissues with differential expression level of FREM1. (A) BC specimens were grouped into low‐ and high‐FREM1 subgroup according to IHC score of FREM1 (low‐FREM1 group, IHC score < 4; high‐FREM1 group, IHC score ≥ 4), and then, subject to IHC for CD4^+^, CD8^+^, and CD68^+^ cells (brown staining). The results are displayed for representative cases with FREM1^low^ or FREM1^high^ BC tissues. Scatter dot plots showing the quantitative analysis of (B) CD4^+^, (C) CD8^+^, and (D) CD68^+^ cells in FREM‐low and FREM1‐high group. Mean ± SD is denoted in the scatter plots. *N* = 19 and *N* = 11 for FREM1‐low and FREM1‐high group, respectively. *p < 0.05, **p < 0.01

**Figure 9:**
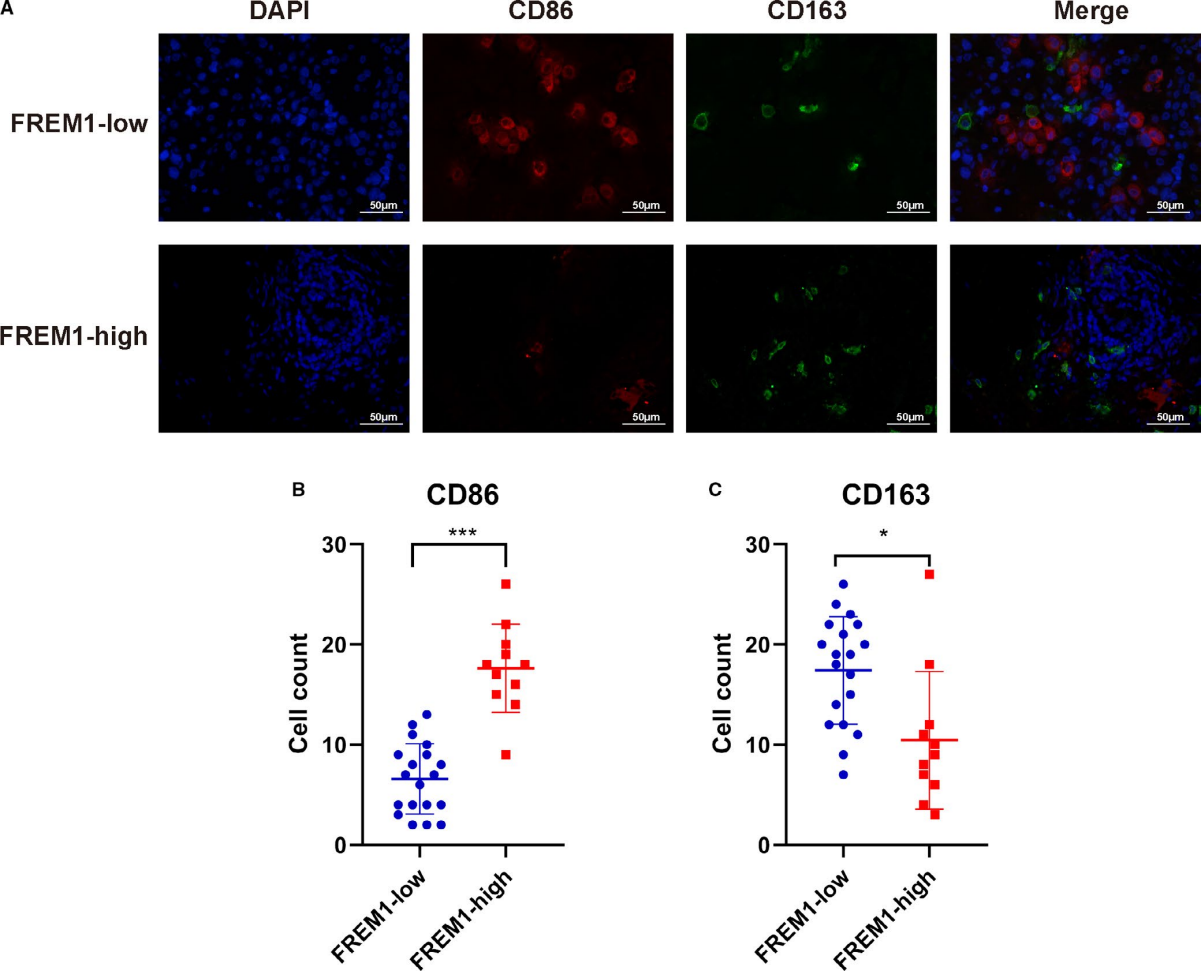
M1‐ and M2‐polarized macrophages in BC tissues with different expression of FREM1. (A) BC tissues were first divided into low‐ and high‐FREM1 group as described previously. IF was conducted to assess the presence of CD86^+^ CD163^−^ M1‐polarized and CD86^−^ CD163^+^ M2‐polarized macrophages in low‐ and high‐FREM1 group. Simples were double stained for CD86‐Alexa Fluor‐647 (red) and CD163‐Alexa Fluor‐488 (green). DAPI represents nuclei of the cells (blue). Images are shown individually and as an overlay of the fluorescence channels on the far right. Scatter plots displaying the quantitative data of (B) CD86^+^ and (C) CD163^+^ cells in FREM‐low and FREM1‐high group. Solid lines in the scatter plots delineate the mean ± SD. *p < 0.05, ***p < 0.001

To further validate the result of CIBERSORT indicating a relationship between FREM1 and immune cell enrichment, we conducted IHC and IF on 30 BC tissue specimens. According to IHC score, simples were categorized into a low‐FREM1 expression group (IHC score < 4, *n* = 19) and high‐FREM1 expression group (IHC score ≥ 4, *n* = 11). First, we evaluated CD68^+^ cells that represent pan‐macrophages and CD4^+^ and CD8^+^ T cells by IHC. Remarkably, a similar result to the CIBERSORT was observed that the number of CD68^+^ macrophages in high‐FREM1 group (47–172, median 91 cells/field) was obviously lower than in low‐FREM1 group (29–239, median 159 cells/field; Figure 8A,B). Whereas the number of CD4^+^ and CD8^+^ T cells in high‐FREM1 group (46–209, median 97 cells/field and 29–139, median 91 cells/field, respectively) was significantly higher than in low‐FREM1 group (13–159, median 33 cells/field and 11–108, median 41 cells/field, respectively; Figure 8A,C,D). Next, we went further to assess the differential distribution of M1‐ and M2‐polarized macrophages between high‐ and low‐FREM1 expression group by double‐stain IF. CD86 is considered as a surface marker of M1 macrophages, whereas CD163 is proposed as a classically surface marker of M2 macrophages. As expected, the numbers of infiltrating CD86^+^ macrophages from high‐FREM1 group were evidently higher than those in low‐FREM1 group (9–26, median 18 cells/field vs. 2–13, median 7 cells/field, high vs. low; Figure 9A,B). In contrast, CD163^+^ macrophages were more frequently observed in low‐FREM1 group than in high‐FREM1 group (3–27, median 9 cells/field vs. 7–26, median 19 cells/field, high vs. low; Figure 9A,C). These results were dovetail with those predicted from CIBERSORT and strongly suggested that FREM1 is intimately linked to the infiltration of immune cells in BC tissues.

## 4 DISCUSSION

FREM1, also known as QBRICK, is a secreted matrix‐related protein encoded by the FREM1 gene located at the chromosome 9p22.3.^10^ A ternary complex formed by it with FRAS1 and FREM2 from the same family is responsible for maintaining the stability of the cell basement membrane.^30^ The cell basement membrane is a thin, compact sheet of ECM that plays a key role in tissue development and functional maintenance. Therefore, abnormalities in the mechanical and chemical properties of the basement membrane are often associated with multiple diseases, particularly cancers. Indeed, two other genes in the FRAS/FREM family, FRAS1 and FREM2, have been shown to be related with the occurrence and progression of certain carcinomas. For instance, Umeda et al. has reported that FRAS1 knockout can inhibit the proliferation and invasion of gastric cancer cell lines through the EGFR and PI3K signaling pathways both in vitro and in vivo.^31^ Similarly, Jovcevska et al. has found a positive relation between the expression of FREM2 and the favorable prognosis of patients with isocitrate dehydrogenase (IDH)‐wild‐type glioblastoma.^32^ Nevertheless, no study on the involvement of FREM1 in carcinoma has been reported.

In the present study, we found that FREM1 expression was significantly decreased in BC tissues compared with that in adjacent non‐tumor tissues by bioinformatics and IHC. Moreover, the methylation of FREM1 in the promoter region of BC was significantly higher than that of normal tissue. Since hypermethylation of the promoter region is associated with dysregulation of gene expression,^33^ it might be inferred that decreased expression of FREM1 could partly be attributed to a consequence of promoter hypermethylation. Moreover, the patients with elderly age, advanced clinical stage and distant metastases exhibited dramatically lower expression of FREM1, heralding the possibility of becoming a more aggressive phenotype for breast tumors with aberrant reduced FREM1 expression. Furthermore, the relationship between the FREM1 expression and the status of three classical BC molecular markers revealed that FREM1 expression was repressed in ER/PR‐, Her‐2^+^ BC, or TNBC. TNBC being highly aggressive could be considered to have less favorable prognosis compared to other BC types.^34^ Therefore, these results suggested that low‐expression level of FREM1 was closely associated with poor clinicopathological characteristics and molecular typing. Much importantly, Kaplan–Meier curves reflected that low expression of FREM1 was correlated with worse OS and RFS of BC patients, and this association also remained significant for OS in the univariate and multivariate models. Of note, the HR value for FREM1 (HR = 0.31) in the multivariate model was distantly farther from 1 than for clinical stage (HR = 1.98). Collectively, these highlight the unique advantages of FREM1 expression outperforming the classic TNM staging for the evaluation of BC survival.

Although aberrant expression of FREM1 was found to be associated with the prognosis and clinicopathological features of BC patients, the mechanism behind it remained unclear. Because the hallmark feature of FRAS/FREM family is the 12 consecutive repeated chondroitin sulfate proteoglycan (CSPG) domain,^35^ which can interact with multiple molecules in the tumor microenvironment and convey anti‐ or pro‐cancer effects depending on the different tumor types.^36^, ^37^ Accordingly, we speculated that the mechanism of FREM1 involving in cancer metabolism may partially be ascribed to the CSPG domain. The results of GSEA demonstrated that the pathways enriched in the low‐FREM1 expression group are related to cell metabolism, protein synthesis and folding, and cancer‐related signaling, therefore, deletion of this gene might result in a broad alterations of tumor cell biological behaviors. Surprisingly, we found that numerous signaling pathways associated with immune regulation are clustered in the high‐FREM1 expression group, such as inflammatory response, JAK‐STAT signaling, cytokine‐cytokine receptor interaction, and T cell receptor signaling. These findings indicated that FREM1 may also involve in the reconstruction and regulation of the tumor immune microenvironment, thereby participating in breast cancer progression beyond affecting the tumor cells per se.

Inflammation is recognized as one of the predominant characteristics of cancer. The tumor microenvironment composed of stromal cells, cytokines, and immune cells has been demonstrated to determine the biological function of tumor cells.^38^ It has been reported that important cell populations in the innate and adaptive immune systems are extensively changed in BC tissues.^39^ Therefore, CIBERSORT analysis was performed to assess whether FREM1 has an impact on the leukocyte representation. Indeed, the distribution pattern of immune cells has dramatically been altered between the high‐ and low‐FREM1 expression groups, specifically manifesting a significantly increased proportion of CD4^+^, CD8^+^, and gamma‐delta T cells, B cells, M1 macrophages, resting dendritic cells, resting mast cells, and eosinophils, as well as a pronouncedly decreased proportion of M0 and M2 macrophages, resting natural killer cells, and neutrophils in the high‐FREM1 expression group compared with the low‐FREM1 group. Intriguingly, almost all increased lineages of immune cells accompanied by an elevated FREM1 expression are considered to have antitumor effects and certain types of them are indicative of a favorable prognosis of BC.^40^, ^41^, ^42^ For instance, the CD8^+^ T cells representing the phenotype of lymphocytes with cytotoxicity mediate antitumor functions by inducing cell lysis via the release of IFN‐gamma and Perforin/granzyme B complex, and they act as a reliable immune prognostic marker for the outcome of BC patients.^43^, ^44^ The processes of CD8^+^ T cells activation and maturation are in turn regulated through the cytokines produced by T‐helper 1 (Th1) cells and tumor‐specific antigens processed by dendritic cells.^45^ Our results demonstrated that the synergistic increase of these cells is accompanied by increased expression of FREM1, suggesting that FREM1 may play a crucial role in regulating the functional integrity of adaptive antitumor immune response. Additionally, the infiltration and polarization of macrophages could also be affected by FREM1, as an increased number of M1 macrophages was found in the high‐FREM1 expression group compared to the low‐FREM1 group, but the situation became the opposite for M2 macrophages. It is generally thought that M1 macrophages elicit antitumor signaling while M2 macrophages exert pro‐tumor effects.^46^ This result suggests a moderate effect of FREM1 in antitumor innate immunity.

Further analysis in the different molecular subtypes revealed that the effect of FREM1 on immune infiltration was consistently observed in Luminal and TNBC subtypes and was specific for Her‐2 overexpression subtype. Of note, more pronounced changes of the CD8^+^ T cells between low‐ and high‐FREM1 expression group did occur in TNBC. Interestingly, previous studies have shown that the antitumor effect of CD8^+^ T cells may be more powerful in TNBC. In a research comprising of 1854 BC patients, Baker et al. found independent prognostic value of CD8^+^ T cells for TNBC, but not for hormone receptor positive tumors.^47^ Hence, the moderating effect on the relationship between FREM1 and CD8^+^ T cells in TNBC warrants continued intensive study. In addition, there were no obvious statistical differences of changes in immune cells distribution except for CD8^+^ T cells in Her‐2 overexpression subtype, possibly due to the relatively small sample size to generate reliable statistics.

Additionally, a significant positive correlation between FREM1 expression and CD4^+^, CD8^+^ T cells and CD86^+^ M1‐macrophages as well as a negative correlation between FREM1 expression and CD68^+^ pan‐macrophages and CD163^+^ M2‐macrophages was further verified by IHC and IF on our BC specimens.

A possible explanation for the alteration of immune cells distribution affected by FREM1 could be attributed to the C‐type lectin‐like domain in the C‐terminus of FREM1 protein structure, which is regarded as an immune‐regulatory receptor involving nonspecific and specific tumor immune responses.^48^ Therefore, we speculated that FREM1 may interact with other chemokines or cytokines existing in the tumor microenvironment through this domain, thereby playing a role in recruiting antitumor immune cells homing to the tumor locality. Altogether these findings indicate that high‐FREM1 expression is intensively associated with antitumor immune infiltration in BC.

Finally, we acknowledged some potential limitations in our study. Despite having been validated on a small group of patients by IHC and IF, mainly this research on FREM1 was based on the analysis of high‐throughput sequencing data, which herein need more rigorous confirmation in a larger‐scale simple size. Further investigations are needed to clarify the underlying mechanism of FREM1 in the progression of BC and the regulation of BC immune microenvironment.

## 5 CONCLUSIONS

Taken together, our findings demonstrate that low‐FREM1 expression was closely associated with poor prognosis and was identified to be an independent predictor for BC. As possible mechanisms, reduced FREM1 expression was related to tumor cell metabolism and protein synthesis process, while increased FREM1 expression was associated with high‐level infiltration of antitumor immune cells in BC. However, further studies are required to elucidate the detailed mechanism of immune‐regulation process of FREM1 in BC.

## CONFLICTS OF INTEREST

The authors declare that they have no competing interests.

## AUTHOR CONTRIBUTIONS

Conceptualization, H.L. and Z.Y.; Methodology, Z.L.; Formal Analysis, M.C.; Investigation, H.L. and M.C.; Writing – Original Draft Preparation, H.L. and Z.L.; Writing – Review and Editing, Z.Y. and X.L.; Visualization, G.W.; Funding Acquisition, X.L.

## Supporting information

Table S1‐S4

## Data Availability

All data generated or analyzed during this study are included in this article.
