# Supplementary material for: Elevated expression of FREM1 in breast cancer indicates favorable prognosis and high‐level immune infiltration status
Source: Cancer Med. 2020 Oct 14;9(24):9554–70. doi: 10.1002/cam4.3543 (PMC7774739; doi:10.1002/cam4.3543)
Supplement: Supplementary file 1 — Table S1‐S4 [file CAM4-9-9554-s001.docx]

Table S1. The demographic features and clinicopathological parameters of breast cancer patients from TCGA database.

| Characteristics | Total No. | No. of patients |
| --- | --- | --- |
| Age (year) | 1097 |  |
| >50 |  | 331 |
| ≤50 |  | 766 |
| Histological type | 1056 |  |
| IDC |  | 784 |
| ILC |  | 203 |
| Medullary carcinoma |  | 6 |
| Mucinous carcinoma |  | 17 |
| Other, specify |  | 46 |
| Clinical stage | 1073 |  |
| I |  | 183 |
| II |  | 621 |
| III |  | 249 |
| IV |  | 20 |
| T stage | 1094 |  |
| T1 |  | 281 |
| T2 |  | 635 |
| T3 |  | 138 |
| T4 |  | 40 |
| N stage | 1077 |  |
| N0 |  | 516 |
| N1 |  | 364 |
| N2 |  | 120 |
| N3 |  | 77 |
| Distant metastasis | 934 |  |
| M0 |  | 912 |
| M1 |  | 22 |
| ER status | 984 |  |
| Negative |  | 228 |
| Positive |  | 756 |
| PR status | 981 |  |
| Negative |  | 325 |
| Positive |  | 656 |
| Her-2 status | 687 |  |
| Negative |  | 532 |
| Positive |  | 155 |
| Classical molecular subtype | 719 |  |
| Luminal |  | 566 |
| Her-2 overexpression |  | 37 |
| TNBC |  | 116 |

TCGA, The Cancer Genome Atlas; IDC, infiltrating ductal carcinoma; ILC, infiltrating lobular carcinoma; ER, estrogen receptor; PR, progesterone receptor; Her-2, human epidermal growth factor receptor-2; TNBC, triple negative breast cancer.

Table S2. Characteristics of GEO series included in this study.

| GEO series | Contributor(s) | N | T | Platform |
| --- | --- | --- | --- | --- |
| GSE71053 | Thomassen M, 2018 | 12 | 6 | Affymetrix Human Genome U133 Plus 2.0 Array |
| GSE120129 | Ernlund A, 2018 | 78 | 30 | Affymetrix Human Genome U133 Plus 2.0 Array |
| GSE42568 | Clarke C, 2013 | 17 | 104 | Affymetrix Human Genome U133 Plus 2.0 Array |
| GSE29431 | Cuadros M, 2011 | 12 | 54 | Affymetrix Human Genome U133 Plus 2.0 Array |
| GSE50567 | Lisowska K, 2011 | 6 | 35 | Affymetrix Human Genome U133 Plus 2.0 Array |

GEO, Gene Expression Omnibus; T, tumor; N, normal.

Table S3. Gene set enriched in the high FREM1 mRNA expression phenotype.

| Gene set | SIZE | ES | NES | NOM  p-val | FDR  q-val |
| --- | --- | --- | --- | --- | --- |
| HALLMARK pathways |  |  |  |  |  |
| HALLMARK_UV_RESPONSE_DN | 142 | 0.618304 | 2.289652 | 0 | 0.002079 |
| HALLMARK_IL2_STAT5_SIGNALING | 200 | 0.562727 | 2.145946 | 0.001969 | 0.006443 |
| HALLMARK_INFLAMMATORY_RESPONSE | 200 | 0.615114 | 2.071458 | 0.005803 | 0.011638 |
| HALLMARK_IL6_JAK_STAT3_SIGNALING | 87 | 0.645236 | 2.021609 | 0.00996 | 0.015611 |
| HALLMARK_TNFA_SIGNALING_VIA_NFKB | 199 | 0.610292 | 1.957763 | 0.022449 | 0.0195 |
| HALLMARK_APICAL_JUNCTION | 200 | 0.527686 | 1.956728 | 0.002028 | 0.01739 |
| HALLMARK_MYOGENESIS | 200 | 0.523297 | 1.912287 | 0 | 0.021561 |
| KEGG pathways |  |  |  |  |  |
| KEGG_JAK_STAT_SIGNALING_PATHWAY | 155 | 0.63857 | 2.403987 | 0 | 0 |
| KEGG_CYTOKINE_CYTOKINE_RECEPTOR_INTERACTION | 264 | 0.64136 | 2.282651 | 0.002088 | 4.84E-04 |
| KEGG_T_CELL_RECEPTOR_SIGNALING_PATHWAY | 108 | 0.671261 | 2.206901 | 0 | 7.81E-04 |
| KEGG_LEUKOCYTE_TRANSENDOTHELIAL_MIGRATION | 116 | 0.582284 | 2.07268 | 0.00202 | 0.005222 |
| KEGG_FC_EPSILON_RI_SIGNALING_PATHWAY | 79 | 0.542774 | 2.032714 | 0.002 | 0.007869 |
| KEGG_ETHER_LIPID_METABOLISM | 33 | 0.5901 | 2.028199 | 0.001969 | 0.007564 |
| KEGG_ASTHMA | 28 | 0.790382 | 2.023923 | 0.005988 | 0.007578 |
| KEGG_DILATED_CARDIOMYOPATHY | 90 | 0.52559 | 1.996578 | 0.002033 | 0.009532 |
| KEGG_MAPK_SIGNALING_PATHWAY | 267 | 0.469546 | 1.990977 | 0 | 0.009542 |
| KEGG_INTESTINAL_IMMUNE_NETWORK_FOR_IGA_PRODUCTION | 46 | 0.775766 | 1.973312 | 0.008032 | 0.010595 |
| KEGG_TIGHT_JUNCTION | 132 | 0.458504 | 1.972132 | 0.00396 | 0.010101 |
| KEGG_HYPERTROPHIC_CARDIOMYOPATHY_HCM | 83 | 0.516069 | 1.964975 | 0.00202 | 0.010533 |
| KEGG_TGF_BETA_SIGNALING_PATHWAY | 85 | 0.531632 | 1.955266 | 0 | 0.010717 |
| KEGG_CHEMOKINE_SIGNALING_PATHWAY | 188 | 0.55393 | 1.95407 | 0.005917 | 0.010344 |
| KEGG_AUTOIMMUNE_THYROID_DISEASE | 50 | 0.710383 | 1.923342 | 0.002037 | 0.01311 |
| KEGG_MELANOMA | 71 | 0.497502 | 1.921317 | 0 | 0.01288 |
| KEGG_PATHWAYS_IN_CANCER | 325 | 0.459832 | 1.915297 | 0.002012 | 0.013273 |
| KEGG_ADIPOCYTOKINE_SIGNALING_PATHWAY | 67 | 0.541853 | 1.914959 | 0.002024 | 0.012851 |
| KEGG_BASAL_CELL_CARCINOMA | 55 | 0.522608 | 1.907604 | 0.006397 | 0.013175 |
| KEGG_LEISHMANIA_INFECTION | 70 | 0.658257 | 1.891644 | 0.018367 | 0.014886 |
| KEGG_ALDOSTERONE_REGULATED_SODIUM_REABSORPTION | 42 | 0.553127 | 1.890808 | 0 | 0.01458 |
| KEGG_ARRHYTHMOGENIC_RIGHT_VENTRICULAR_CARDIOMYOPATHY_ARVC | 74 | 0.497137 | 1.864627 | 0 | 0.01792 |
| KEGG_RENAL_CELL_CARCINOMA | 70 | 0.500252 | 1.853406 | 0.006 | 0.018675 |

FREM1, FRAS1 Related Extracellular Matrix 1; ES, enrichment score; NES, normalized enrichment score; NOM, nominal; FDR, false discovery rate.

Table S4. Gene set enriched in low FREM1 mRNA expression phenotype.

| Gene set | SIZE | ES | NES | NOM  p-val | FDR  q-val |
| --- | --- | --- | --- | --- | --- |
| HALLMARK pathways |  |  |  |  |  |
| HALLMARK_GLYCOLYSIS | 200 | -0.65159 | -2.47437 | 0 | 0 |
| HALLMARK_MYC_TARGETS_V1 | 199 | -0.84016 | -2.38294 | 0 | 0 |
| HALLMARK_OXIDATIVE_PHOSPHORYLATION | 200 | -0.8094 | -2.33935 | 0 | 4.91E-04 |
| HALLMARK_MTORC1_SIGNALING | 200 | -0.70034 | -2.26973 | 0 | 5.63E-04 |
| HALLMARK_E2F_TARGETS | 200 | -0.82744 | -2.22768 | 0.002096 | 8.88E-04 |
| HALLMARK_MYC_TARGETS_V2 | 58 | -0.85047 | -2.21351 | 0 | 8.17E-04 |
| HALLMARK_DNA_REPAIR | 150 | -0.66995 | -2.20285 | 0 | 7.64E-04 |
| HALLMARK_G2M_CHECKPOINT | 199 | -0.68858 | -2.05593 | 0.00823 | 0.003195 |
| KEGG pathways |  |  |  |  |  |
| KEGG_HUNTINGTONS_DISEASE | 180 | -0.68827 | -2.37916 | 0 | 0 |
| KEGG_OXIDATIVE_PHOSPHORYLATION | 131 | -0.81039 | -2.36108 | 0 | 0 |
| KEGG_PARKINSONS_DISEASE | 127 | -0.74749 | -2.22069 | 0 | 0.001123 |
| KEGG_AMINOACYL_TRNA_BIOSYNTHESIS | 41 | -0.77203 | -2.21128 | 0 | 0.001108 |
| KEGG_PYRIMIDINE_METABOLISM | 98 | -0.61735 | -2.19719 | 0 | 0.001507 |
| KEGG_ALZHEIMERS_DISEASE | 165 | -0.62857 | -2.19699 | 0 | 0.001319 |
| KEGG_RNA_POLYMERASE | 29 | -0.76926 | -2.18245 | 0 | 0.001275 |
| KEGG_PROTEIN_EXPORT | 24 | -0.80913 | -2.15365 | 0 | 0.001613 |
| KEGG_DNA_REPLICATION | 36 | -0.84699 | -2.10242 | 0 | 0.003547 |
| KEGG_PYRUVATE_METABOLISM | 40 | -0.63343 | -2.0997 | 0 | 0.003519 |
| KEGG_HOMOLOGOUS_RECOMBINATION | 28 | -0.70615 | -2.08921 | 0.003945 | 0.003578 |
| KEGG_SPLICEOSOME | 127 | -0.66674 | -2.05717 | 0 | 0.00496 |
| KEGG_NUCLEOTIDE_EXCISION_REPAIR | 44 | -0.64558 | -2.04963 | 0.005758 | 0.005269 |
| KEGG_CITRATE_CYCLE_TCA_CYCLE | 31 | -0.75653 | -2.03782 | 0.002062 | 0.005821 |
| KEGG_AMINO_SUGAR_AND_NUCLEOTIDE_SUGAR_METABOLISM | 43 | -0.60325 | -2.00828 | 0.004057 | 0.007364 |
| KEGG_VIBRIO_CHOLERAE_INFECTION | 54 | -0.59413 | -2.00522 | 0.002137 | 0.007156 |
| KEGG_BASE_EXCISION_REPAIR | 35 | -0.71403 | -2.0032 | 0.003937 | 0.006938 |
| KEGG_MISMATCH_REPAIR | 23 | -0.75202 | -1.96502 | 0.007952 | 0.010151 |
| KEGG_TERPENOID_BACKBONE_BIOSYNTHESIS | 15 | -0.76345 | -1.92754 | 0.008163 | 0.012769 |
| KEGG_BIOSYNTHESIS_OF_UNSATURATED_FATTY_ACIDS | 22 | -0.67555 | -1.92394 | 0.00611 | 0.012482 |
| KEGG_PENTOSE_PHOSPHATE_PATHWAY | 27 | -0.62108 | -1.91983 | 0.006036 | 0.012389 |
| KEGG_CARDIAC_MUSCLE_CONTRACTION | 78 | -0.52262 | -1.88573 | 0.007692 | 0.014433 |
| KEGG_ALANINE_ASPARTATE_AND_GLUTAMATE_METABOLISM | 32 | -0.54569 | -1.88151 | 0.00409 | 0.014205 |
| KEGG_PORPHYRIN_AND_CHLOROPHYLL_METABOLISM | 41 | -0.6091 | -1.88073 | 0.004008 | 0.013788 |
| KEGG_RNA_DEGRADATION | 59 | -0.53937 | -1.82513 | 0.02008 | 0.02152 |
| KEGG_GLYCOLYSIS_GLUCONEOGENESIS | 62 | -0.5247 | -1.82228 | 0.004124 | 0.02123 |

FREM1, FRAS1 Related Extracellular Matrix 1; ES, enrichment score; NES, normalized enrichment score; NOM, nominal; FDR, false discovery rate.


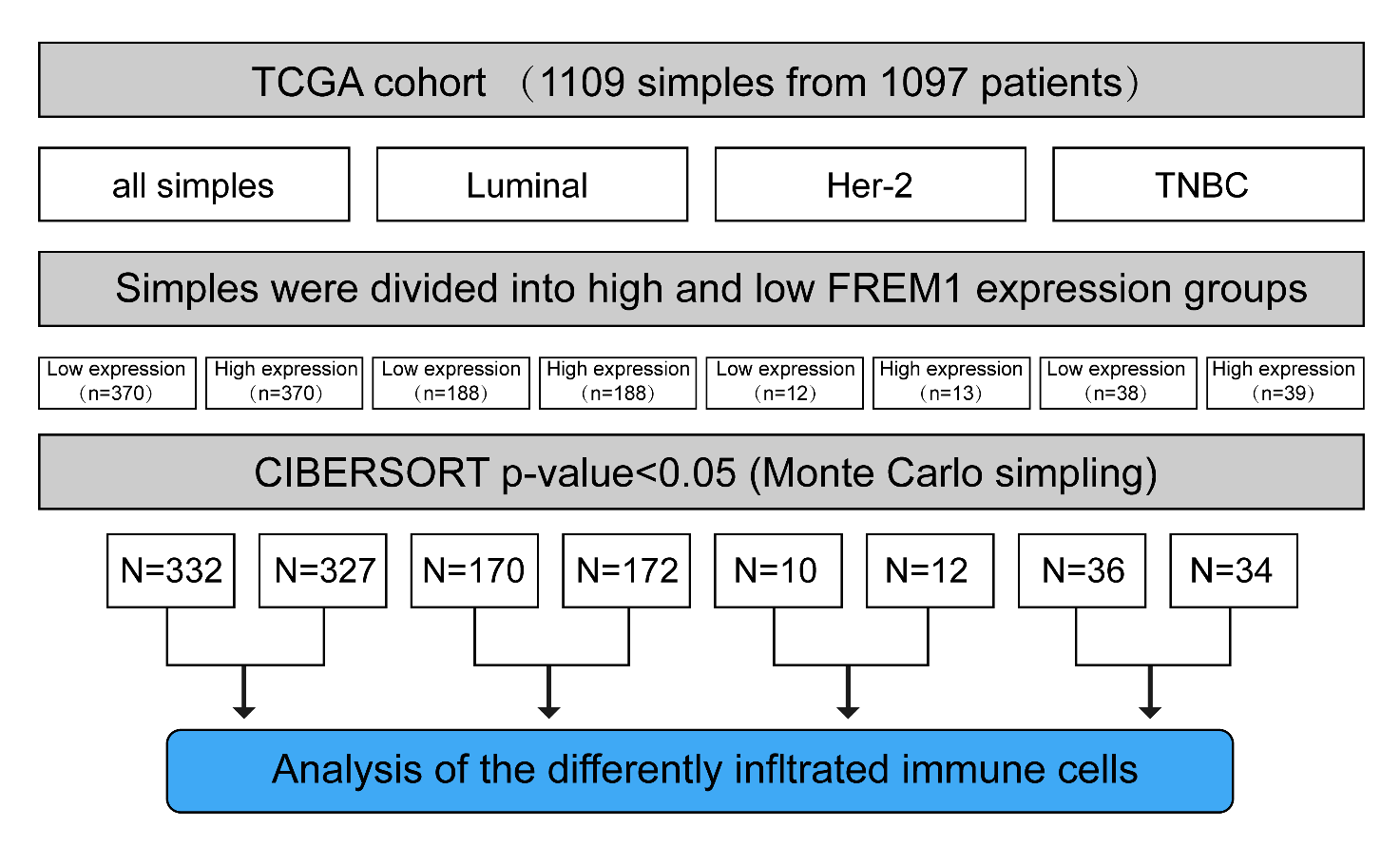


Figure S1. Flowchart detailing the CIBERSORT design.


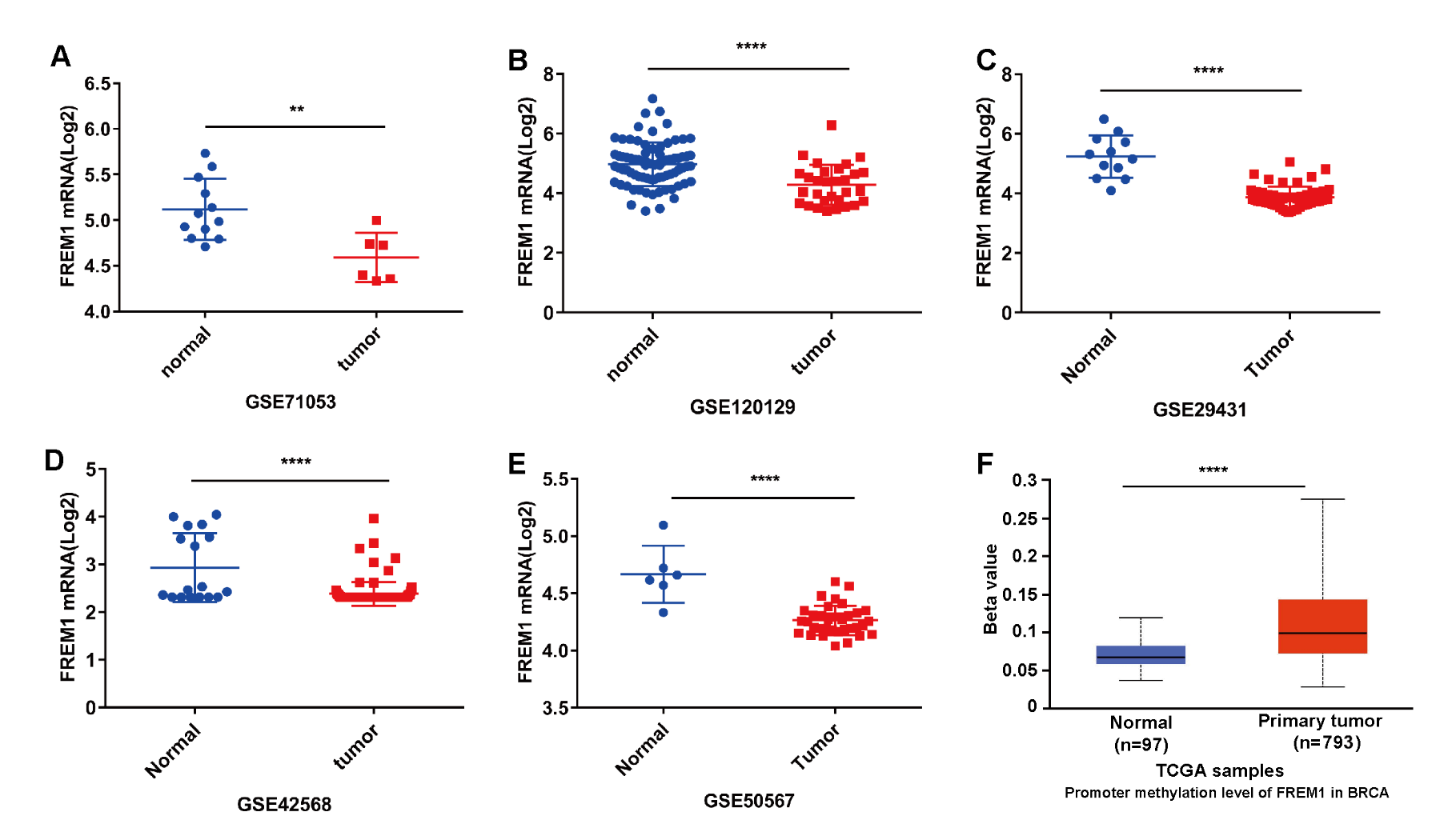


Figure S2. FREM1 transcriptional expression and promoter methylation. (A-E) Dot plots showing the FREM1 mRNA levels in GSE71053, GSE120129, GSE42568, GSE29431, and GSE50567, respectively. (F) Box plot showing the FREM1 promoter methylation levels of BC and adjacent normal tissues in TCGA. **p<0.01, ****p<0.0001. FREM1, FRAS1 Related Extracellular Matrix 1; BC, breast cancer; TCGA, The Cancer Genome Atlas.
